# Enhancing the Compatibility of Poly (1,4-butylene adipate) and Phenoxy Resin in Blends

**DOI:** 10.3390/ma10070692

**Published:** 2017-06-23

**Authors:** Cheng-Fu Yang, Hsiang-Ching Wang, Chean-Cheng Su

**Affiliations:** Department of Chemical and Materials Engineering, National University of Kaohsiung, No. 700, Kaohsiung University Rd., Nan-Tzu Dist., Kaohsiung 811, Taiwan; cfyang@nuk.edu.tw (C.-F.Y.); celestine772000@yahoo.com.tw (H.-C.W.)

**Keywords:** compatibility, alcoholytic exchange, glass transition temperature, interaction parameter, homogeneous phase

## Abstract

This work concerns the enhancement in the compatibility of blends of poly (1,4-butylene adipate) (PBA) with poly (hydroxy ether of bisphenol-A) (phenoxy) via alcoholytic exchange. Results on the thermal behavior and morphology show that the blended PBA/phenoxy system exhibits a homogeneous phase and a composition-dependent glass transition temperature (T_g_). The interaction parameter (*χ*_12_) of PBA/phenoxy blends was calculated using the melting point depression method and was found to be −0.336. However, the compatibilization of PBA/phenoxy blends can be enhanced by chemical exchange reactions between PBA and phenoxy upon annealing. Annealed PBA/phenoxy blends were found to have a homogeneous phase with a higher T_g_ than that of the blended samples, and a smooth surface topography that could be improved by annealing at high temperature. The results of this investigation demonstrate that promotional phase compatibilization in the PBA/phenoxy blend can only be obtained upon thermal annealing, thus causing transreactions to occur between the dangling –OH of the phenoxy and the ester functional groups in PBA. Extensive transreactions cause alcoholytic exchange between the PBA and phenoxy to form a network, thus reducing the mobility of the polymer chain. Finally, the crystallinity of PBA decreased as the degree of transreaction in the blends increased.

## 1. Introduction

Polymer blending is a more economical method for making new materials or compounds compared to direct polymer synthesis. Blending several polymers with various properties may be a unique way to develop new materials with flexible compositions containing various constituents, thus offering flexibility and property-balancing [1,2,3,4,5,6,7,8,9,10,11,12,13]. Rana et al. [7,8,9,10,11,12,13] reported a series miscible or immiscible blends such as poly (phenyl acrylate)/poly (styrene-co-acrylonitrile), poly (styrene-co-acrylonitrile)/poly (vinyl benzoate), blends of ethylene 1-octene copolymers, poly (vinyl ester)s/polyacrylates, and polyethylene/polyolefins blends that result in novel properties for various end uses. Various factors affect the properties of polymer blends, including the combined polymer species, modifiers, solvent, catalysts, blending temperature, molecular weight of the polymers, and structures of the molecules and their mutual interactions [14,15,16,17,18].

Miscible blends possess thermodynamics solubility and are characterized by the presence of a single phase and glass transition temperature (T_g_). Their properties can often be predicted from the weighted average composition of the properties of the individual components. To achieve miscibility in polymer blends, a negative free energy of mixing (ΔG = ΔH − TΔS) must exist that, in turn, requires an exothermic heat (ΔH) of mixing because entropic (ΔS) contributions are negligible. An exothermic heat of mixing can be achieved by the introduction of specific interactions between blend components. The potentially useful specific interactions include chemical (strong covalent) and physical interactions (hydrogen bonding, ion–dipole, dipole–dipole, and donor–acceptor interactions) [14,19,20]. However, most polymer pairs are immiscible. Some immiscible blends with phase separation have successfully found commercial applications; for example, high-impact polystyrene and acrylonitrile–butadiene–styrene terpolymer. The key to making successful blends of this type is the use of compatibilization to control morphology that promotes the compatibility of polymer blends. Compatible blends are characterized by the presence of a finely dispersed phase, good adhesion between phases, and technologically desirable properties. There are many methods of compatibilizing immiscible blends, including the introduction of nonreactive grafts or block copolymers, nonbonding specific interactions, coupling agents, and reactive polymers [14].

Reactive polymer blending has become very important in the development of new polymer materials [14]. Many reactive polymers have been used to compatibilize polymer blends. Reactive polymers are classified into seven major categories based on their reactive functionalities including maleic anhydride, carboxylic acid, groups that can undergo interchange reactions, primary and secondary amines, hydroxyl groups, heterocyclic groups, or groups that can undergo ionic interactions. These reactive polymers are used in the compatibilization of various polymer blends to improve their mechanical and electrical properties, thermal behavior, crystallization, and morphology [21,22,23].

**Scheme 1:**
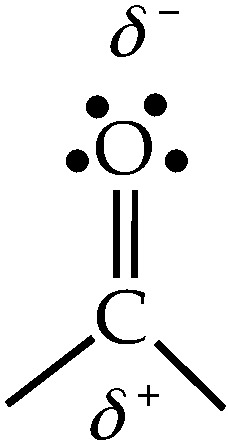
Electron density of the carbonyl group.

Polyester refers to a class of polymers that contains an ester functional group in its main chain. Based on the composition of their main chain, polyesters have an aliphatic structure polycaprolactone (PCL), polybutylene succinate (PBS), polyethylene adipate (PEA), a semi-aromatic structure polyethylene terephthalate (PET), polybutylene terephthalate (PBT), polytrimethylene terephthalate (PTT), polyethylene naphthalate (PEN), or an aromatic structure (liquid-crystal polymers, Kevlar) [22,24,25,26]. An analysis of the chemistry of the carbonyl group reveals that the difference between the electronegativities of carbon and oxygen atoms makes the C=O bond moderately polar. In addition, compounds with a carbonyl group possess hybrid resonance structures. This polar hybrid has a negative charge on the oxygen and a positive charge on the carbon of the C=O double bond. Scheme 1 shows the electron density of the carbonyl group. The carbonyl carbon atom becomes electrophilic and thus reacts with nucleophiles. Simultaneously, the electronegative oxygen atom reacts with an electrophile. In general, chemicals that attack the electron-rich (δ^−^) end of the C=O bond are known as electrophiles, which include ions and neutral molecules that are Lewis acids (electron-pair acceptors). Electrophilic compounds that attack the electron-poor (δ^+^) end of this bond are nucleophiles (Lewis bases) [27,28,29].

Poly (hydroxyl ether of bisphenol-A) (phenoxy) is an amorphous thermoplastic that incorporates pendant hydroxyl groups that are reactive upon heating. The pendant hydroxyl group in the repeating unit enables interaction with proton-accepting functional groups in the polymers. Two interactions that can increase the compatibility of phenoxy and other polymers in polymer blends include hydrogen and covalent bonding. Therefore, phenoxy is often used in polymer blending to modify the polymer compounds [30,31,32,33,34].

In polymer blends, chemical interactions between charge transfer complexes and ionic interactions of ionomers are commonly observed between molecules or segments in a polymer. Condensation polymer blends, such as PBT/phenoxy [26], PET/phenoxy [35], P (BS-co-BT)/phenoxy [21,22], and PHB/phenoxy [34] are immiscible in the blended state but undergo a transreaction during mixing at high temperature, causing separated phases in the blends to become partially or wholly miscible. According to the literature, the main transreaction is alcoholysis. A transreaction or exchange reaction initially forms block copolymers and subsequently forms copolymers with random compositions that improve the miscibility of the blend.

This study investigates compatibilization and chemical interaction in blends of poly (1,4-butylene adipate) (PBA) with poly (hydroxy ether of bisphenol-A) (phenoxy), and the reactive polymer blend has the potential to be used for the development of new polymer materials. PBA was used as a model polymer to blend with phenoxy to elucidate the effects and extent of the chemical interactions and the annealing temperature on miscibility, crystallization, thermal behavior, and morphology of the blends. Furthermore, products of the exchange reaction between PBA and phenoxy were examined in detail.

## 2. Experimental

### 2.1. Materials

**Scheme 2:**
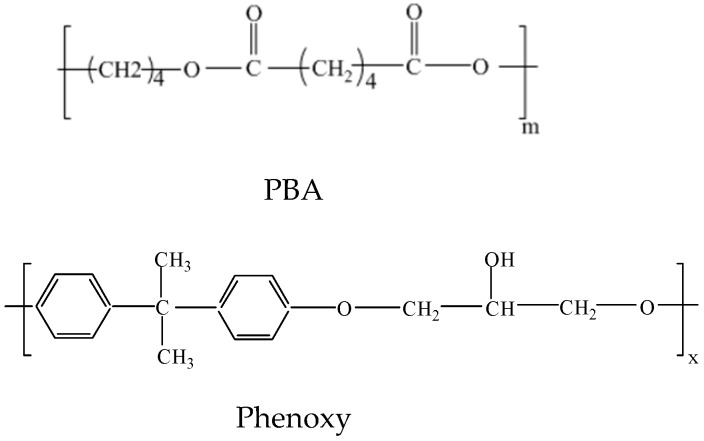
The structures of the repeat units of PBA and phenoxy.

PBA with M_w_ = 12,000 g/mol was purchased from Sigma-Aldrich (Steinheim, Germany). Phenoxy with M_w_ = 60,000 g/mol was purchased from Scientific Polymer Products (New York, NY, USA). The structures of the repeat units of PBA and phenoxy are as Scheme 2.

### 2.2. Preparation of Blends

PBA/phenoxy blends were prepared by solution blending by using methylene chloride as a medium. Polymer solutions were thoroughly stirred and then cast at 40 °C as films on substrates. The solvent in the film samples was allowed to evaporate at room temperature, then degassing was performed for 48 h in a vacuum oven at 80 °C to remove residual solvent from the samples before characterization. The PBA/phenoxy compositions were 10/90, 30/70, 50/50, 70/30, and 90/10. These samples are referred to as “as-blended”. As-blended materials were further held at 260 °C or 280 °C for various times to elucidate the effects of heat annealing. These latter samples are designated as “heat-annealed”. The soluble part of the reacted samples was extracted using methylene chloride and the residual solids and extracted solution were separately kept for Fourier-transform infrared spectroscopy (FT-IR).

### 2.3. Characterization

The thermal behavior of PBA/phenoxy blends was measured using a differential scanning calorimeter (DSC) (Perkin-Elmer PYRIS I, Waltham, MA, USA) equipped with an intracooler under purging with dry nitrogen. For nonisothermal crystallization, melted PBA/phenoxy blends were cooled to 0 °C at a cooling rate of 10 °C/min. The samples underwent the following thermal cycles. Samples were heated rapidly from 0 to 280 °C at which they were held for various annealing times (t_a_), then cooled at 10 °C/min to 0 °C. The annealing times for the PBA/phenoxy blends heated at 280 °C were 2, 4, and 6 min. The blends underwent various numbers of cycles (*n*). The total annealing time ∑t_a_ (280 °C) ≡ n·t_a_ (280 °C). Moreover, ∑t_a_ (280 °C) was increased from 2 (*n =* 1) to 42 min (*n =* 7). FT-IR (Perkin-Elmer Spectrum 100, Waltham, MA, USA) was used to identify functional groups in the blends and possible interactions between PBA and phenoxy. Spectra were obtained with a resolution of 4 cm^−1^, and their averages were obtained from at least 64 scans in the standard wavenumber range of 400–4000 cm^−1^. The blends were solution-cast as thin and uniform films onto KBr pellets which then underwent necessary thermal treatments. Vacuum-dried KBr-cast film samples were used for making IR measurements at ambient temperatures. The morphology of the PBA/phenoxy blends was examined using a scanning electron microscope (Hitachi HF-2000, Tokyo, Japan). The fractured samples were coated with platinum by vapor deposition using a vacuum sputter. Some of the fractured samples were etched using methylene chloride before they were sputter-coated and examined using a microscope. Atomic force microscopy (AFM) was conducted in an intermittent tapping mode by using a diCaliber (Veeco, Santa Barbara, CA, USA) with a silicon tip (*ν* = 70 kHz, *r* = 10 nm). AFM software was used to obtain a height profile along a marked line.

## 3. Results and Discussion

### 3.1. Thermal Analysis

**Figure 1:**
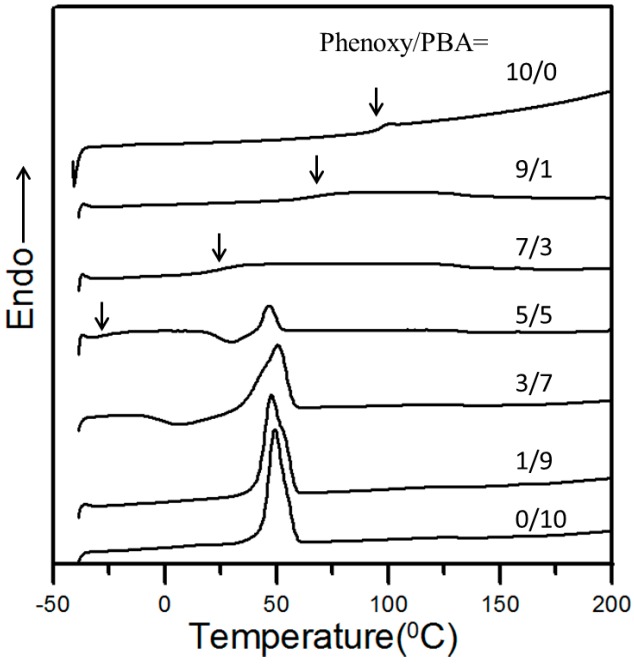
Differential scanning calorimeter (DSC) traces for quenched PBA/phenoxy blends.

In the compatibility test of the polymer blends, a single glass transition temperature (T_g_) value for the PBA/phenoxy blends was used to identify the miscibility during DSC experiments [14,36,37,38]. Amorphous samples that were pre-melted and quenched were characterized to determine the T_g_ Figure 1 plots the T_g_ of PBA/phenoxy blends; those with low PBA content had only a single, composition-dependent T_g_. The T_g_ of the PBA-rich compositions (PBA > 70 wt %) was almost nondetectable because of the inability of PBA to be quenched into a state of sufficiently amorphous phase fractions. The T_g_ of the blended samples sequentially increased with the weight percentage of phenoxy (high-Tg component) in the blend. As the T_g_ of the blends increased, the crystallization window of PBA became narrow; thus, certain phenoxy contents could not crystallize. The DSC results reveal that the crystal melting peak from PBA completely disappeared when the phenoxy content in the blend exceeded 70 wt %. The DSC results suggested that PBA can be mixed homogeneously with phenoxy to form a miscible blend. During the heating of blends with high PBA content, crystallization occurs between the T_g_ and the crystalline-melting temperature (T_m_), as shown by the crystallization exotherm and melt endotherm of PBA in DSC traces. The high PBA-rich compositions (phenoxy/PBA = 10/90, 30/70, and 50/50) have a melting point of 50 °C because of dilution and hindrance of phenoxy in the PBA/phenoxy blends. In the temperature scanning experiment, the crystallization rate was proportional to the PBA content of the PBA/phenoxy blends. The crystalline-melting endotherm of PBA was much lower for PBA-rich PBA/phenoxy blends than for neat PBA, which is a typical result caused by less structural perfection in the polymer crystallites.

**Figure 2:**
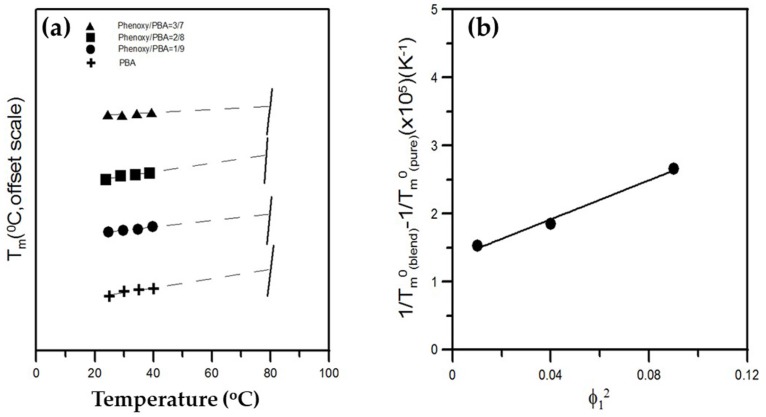
(**a**) Hoffman–Weeks plots for PBA/phenoxy blends (100/0, 90/10, 80/20, and 70/30) crystallized at various Tc and (**b**) Flory–Huggins plot for interaction parameters.

Flory–Huggins thermodynamic analysis is widely used to elucidate favorable interactions of polymer blends based on a reduction in the melting point of the crystalline polymer when two components of a miscible mixture interact with each other [39,40]. The Hoffman–Weeks method [41] was used to obtain the equilibrium melting temperatures of PBA in the PBA/phenoxy blends. In this method, the measured T_m_ of the specimens that were crystallized at T_c_ was plotted against T_c_. A linear extrapolation to the line T_m_ = T_c_ was performed and the intercept of the line yielded T_m_. Specimens were heated at 20 °C/min to a pre-melting temperature of 80 °C where they were held for 5 min to erase the thermal history. Next, the samples were quenched to the desired crystallization temperatures (T_c_s) at a cooling rate of 320 °C/min and held at that temperature until crystallization was complete. The samples were finally scanned at a rate of 10 °C/min. PBA/phenoxy blends were melt-crystallized at various temperatures for 8 h. The desired T_c_s values were selected in the range between 25 and 40 °C. Four PBA/phenoxy blends (neat PBA, 90/10, 80/20, and 70/30) were used. The melting temperatures were used to determine the equilibrium melting temperature. Figure 2a shows Hoffman-Weeks plots for PBA/phenoxy blends (100/0, 90/10, 80/20, and 70/30) that were crystallized at various T_c_, indicating that the equilibrium temperature decreased as the amorphous phenoxy content in the blends increased. The equilibrium melting temperatures of neat PBA and PBA/phenoxy blends (90/10, 80/20, and 70/30) were 80.1 °C, 79.1 °C, 77.6 °C, and 77.4 °C, respectively.

The classical method of reducing the melting point to estimate the Flory-Huggins interaction parameter (*χ*_12_) between PBA and phenoxy was used as follows in Equation (1) [41].

(1) $$\frac{1}{T_{m}}-\frac{1}{T_{m}^{0}}=-\frac{R\nu_{2}}{\Delta H_{f_{2}}\nu_{1}}\chi_{12}{\left(1-\phi_{2}\right)}^{2}$$

where *T_m_* and *T_m_* represent the equilibrium melting points of PBA in the blends and the neat crystallizing polymer, respectively. The subscript “1” refers to the phenoxy, whereas subscript “2” refers to the crystallized polymer PBA. Moreover, *v*_1_ and *v*_2_ are the molar volumes of the repeat units of the phenoxy and PBA, respectively. $\Delta H_{f2}$ is the heat of fusion of the fully crystalline PBA, and *φ*_2_ is the volume fraction of the PBA. The interaction parameter *χ*_12_ was evaluated from the slope of the plot of the left-hand-side of Equation (1) against the right-hand-side of Equation (1). The physical constants that were used in the calculations were *v*_1_ = 242.4 cm^3^·mol^−1^, *v*_2_ = 36.4 cm^3^·mol^−1^, and $\Delta H_{f_{2}}$ = 5.25 KJ·mol^−1^ [42,43]. Figure 2b plots ($\frac{1}{T_{mb}}-\frac{1}{T_{m}^{0}}$) vs. $\phi_{1}^{2}$, which was linearized using the least-square method. A linear equation was used to fit the experimental data, which was calculated from Hoffman–Weeks plots for the PBA/phenoxy blends. A smaller residual sum of squares represents a regression function that explains a greater amount of the data. The residual sum of squares is 0.007 for fitting the Flory–Huggins plot for interaction parameters. The slope of the line yields *χ*_12_ = −0.336, indicating favorable interactions. The negative value of *χ*_12_ reveals that the PBA/phenoxy blend is asec3dot3-materials-10-00692miscible system; this finding is consistent with the characterization of the thermal transition in the DSC experiments.

### 3.2. Specific Interactions upon Annealing

**Figure 3:**
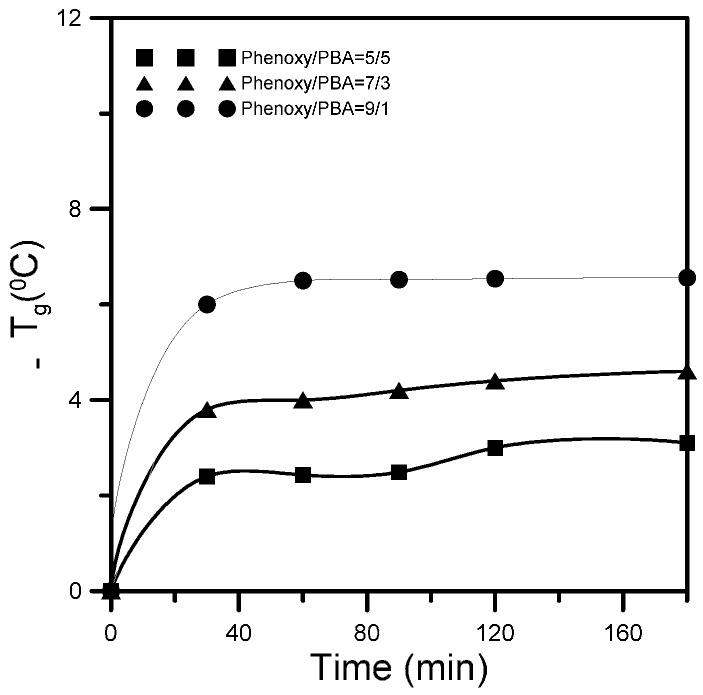
Glass transition temperatures versus mixture compositions after being isothermally treated at 260 °C for 180 min.

To determine the increase in T_g_ as a function of the duration of heating of the blends, the T_g_ values of PBA/phenoxy blends that were heated to 260 °C for various times were determined. Figure 3 plots the increase of T_g_ of the blends, ΔT_g_ (the difference between the T_g_ values of the heat-annealed and as-blended samples), as a function of heating time at 260 °C. The figure reveals that T_g_ increases with the duration of heating of the PBA/phenoxy blends (50/50, 30/70, and 10/90). The T_g_ values of the isothermally treated PBA/phenoxy blends significantly exceed those of the as-blended mixtures. In the annealing treatment of PBA/phenoxy blends, the increase in ΔT_g_ was almost complete within the first 30 min, and the largest increase in ΔT_g_ was exhibited by the blend with PBA/phenoxy = 50/50. For a given duration of heating, the PBA/phenoxy blends that contained more phenoxy had a lower ΔT_g_. T_g_ increases with the duration of heating at 260 °C because the reaction between the components in the blends becomes more extensive; this reaction involves reaction mechanisms that will be discussed in the following sections.

**Figure 4:**
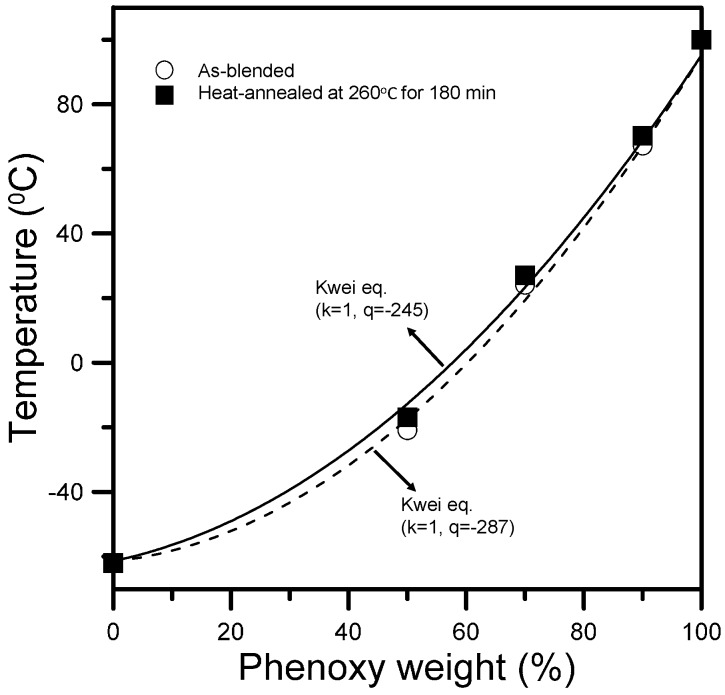
Plots of increase of glass transition temperatures of PBA/phenoxy blends as a function of heating times at 260 °C. The curves show fitting of the data with the Kwei equation.

To further analyze the relationship between T_g_ and composition, the dependence of the T_g_ values of the as-blended and heat-annealed samples on their composition was fitted using models that are based on the thermodynamics of glass transition. Figure 4 plots the experimental T_g_ of the compositions of the as-blended and heat-annealed samples. The single and composition-dependent T_g_ clearly shows that the PBA and phenoxy are completely miscible in PBA/phenoxy blends (50/50, 30/70, and 10/90). The correlation between T_g_ and the composition of as-blended and heat-annealed PBA/phenoxy blends was described by the Kwei equation (Equation (2)) [19,44], which is widely applied for characterizing the T_g_ behavior in polymer blends with specific interactions. The Kwei equation is shown as follows:

(2) $$T_{g}=\frac{w_{1}T_{g1}{+\mathrm{kw}}_{2}T_{g2}}{w_{1}{+\mathrm{kw}}_{2}}{+\mathrm{qw}}_{1}w_{2}$$

where w_1_ and w_2_ are weight fractions of the two components in the blends, T_g1_ and T_g2_ represent the corresponding glass transition temperatures, and k and q are fitting parameters. Note that the first term on the right-hand side of Equation (2) is the widely-used Gordon–Taylor equation, which can be formally derived using the additive rule of the entropy or the volume of the mixtures [4]. Moreover, the second quadratic term qw_1_w_2_ is proportional to the strength of specific interaction in the blend and the balance between the breaking of self-association interactions and the formation of interassociation interactions [19]. Figure 4 plots T_g_ vs. composition, which was fitted using a least-square method. The residual sums of squares are 0.008 and 0.009 for fitting the Kwei equation for interaction parameters of the as-blended and heat-annealed blends of PBA/phenoxy, respectively. In the figure, the following relationships are shown for comparison: k = 1 and q = −245 fitting with the T_g_ data for the heat-annealed blends of PBA/phenoxy, and k = 1 and q = −287 by fitting with the T_g_ data for the as-blended PBA/phenoxy. The negative q value is believed to be attributed to the existence of favorable intermolecular interactions between PBA and phenoxy [4,45]. Based on the q values estimated and demonstrated for as-blended and heat-annealed blends of PBA/phenoxy, the interaction strength in the heat-annealed blends of PBA/phenoxy is stronger than the as-blended of PBA/phenoxy blends. A fitting curve of similar nature has been reported previously for miscible polymer blends with specific interactions [4].

### 3.3. Chemical Interaction in PBA/Phenoxy Blends upon Annealing

**Figure 5:**
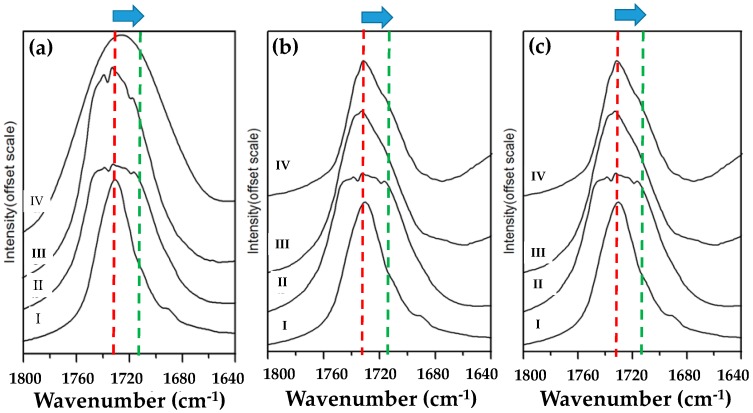
Fourier-transform infrared spectroscopy (FT-IR) spectra of PBA/phenoxy blends in the carbonyl stretching region for blends annealed at 260 °C for 180 min. (**a**) PBA/phenoxy = 50/50; (**b**) PBA/phenoxy = 70/30; and (**c**) PBA/phenoxy = 90/10. In each diagram, spectrum (I) is the as-blended, (II) is the extracted solution, (III) is the heat-annealed sample, and (IV) is the leached solid sample, respectively.

The results of thermal analyses suggest that some chemical reactions might have occurred as a result of the heating treatment of the PBA/phenoxy blend. FT-IR was used to identify the species in the molecules that may participate in chemical interactions in PBA/phenoxy blends. Figure 5a–c show, from top to bottom, carbonyl absorption peaks from four sets of samples that were prepared from the PBA/phenoxy (50/50, 70/30, and 90/10) blends that were annealed at 260 °C for 180 min. For each composition, the carbonyl absorbance shifts in the IR spectra of the samples of the extracted solute (curve II) and leached solid (curve IV) are shown, along with the spectrum of the heated blends (unleached) that is shown on the same diagram as curve III. The main absorption peak of the carbonyl group is at 1735 cm^−1^. One of our earlier studies demonstrated that transreactions or alcoholysis exchange (between polyester and phenoxy) increase the number of aromatic linkages with the carbonyl group, thus downshifting the carbonyl IR absorption peak [2,4]. Table 1 shows the infrared carbonyl band with various linkages adjacent to the carbonyl group. Polymers with various linkages adjacent to the carbonyl group cause a shift in the carbonyl IR absorption. The carbonyl group in the original unreacted PBA chain is linked to two aliphatic linkages; however, transreactions change the linkages adjacent to the carbonyl group. Figure 5 shows that the leached solid sample primarily contains species of the transreaction, polymer segments with aliphatic–(CO)–O–aromatic and aromatic–(CO)–O–aromatic structures. In the heat-annealed PBA/phenoxy blends, extensive transreactions produced a crosslinked network that formed residual solids in the methylene chloride.

**Figure 6:**
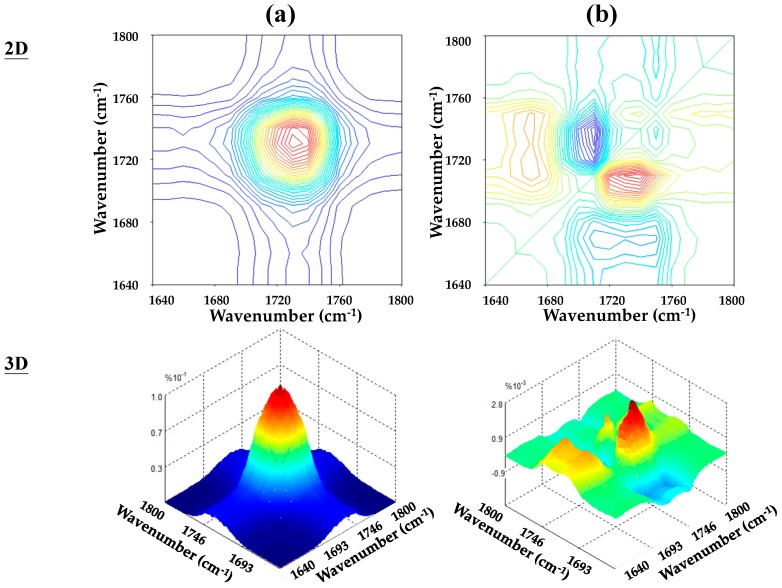
2D and 3D IR analyses: (**a**) synchronous and (**b**) asynchronous correlation spectra for PBA/phenoxy (50/50) blends have been heated at 260 °C for various times.

2D and 3D IR correlation spectroscopy was used to extract more detailed information about the spectral variations that were caused by chemical interactions between PBA and phenoxy. Figure 6a,b show the synchronous and asynchronous correlation spectra in the 1640–1800 cm^−1^ region that were obtained from the time-dependent IR spectra of the PBA/phenoxy (50/50) blend that was annealed at 260 °C. The pair of positive cross peaks at 1732 cm^−1^ reveal that the intensities of the correlated band varied in the same direction. As shown in the Figure 6, the strength of the IR absorption of the polymer segment with aliphatic–(CO)–O–aromatic and aliphatic–(CO)–O–aliphatic structures increases with the annealing time of the blend. In the asynchronous spectrum (Figure 6b), three pairs of cross peaks at 1732, 1738, and 1742 cm^−1^ were observed. The presence of asynchronous cross peaks shows out-of-phase changes in the intensities of the correlated bands.

Figure 6b shows that the heat-annealed samples exhibited an asynchronous correlation with carbonyl absorption bands at 1732, 1738, and 1742 cm^−1^, attributed to the C=O bands of the aromatic–(CO)–O–aromatic, aliphatic–(CO)–O–aliphatic, and aliphatic–(CO)–O–aromatic structures, respectively. The positive sign of the asynchronous cross peaks in the asynchronous spectra reveals that the amount of aliphatic–(CO)–O–aromatic and aliphatic–(CO)–O–aliphatic structures in the blend depends on the annealing time.

### 3.4. Chemical Exchange Reactions in PBA/Phenoxy Blends

**Scheme 3:**
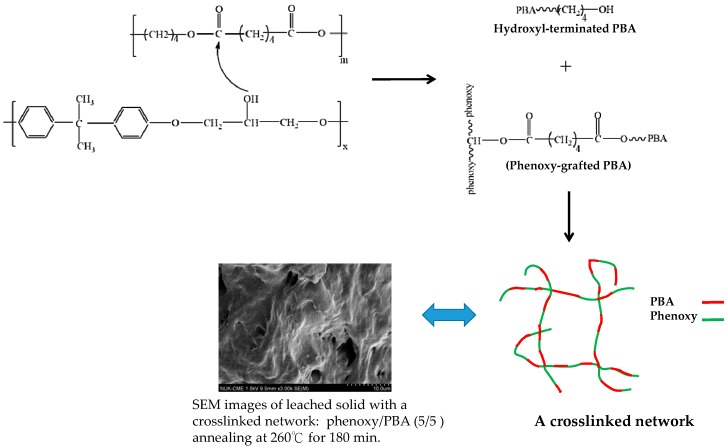
Mechanisms of the chemical exchange reactions between phenoxy and PBA.

An earlier study found that favorable interchange reactions that involve the −OH and carbonyl groups may proceed in aliphatic polyesters and phenoxy blends [2,21,22]. Owing to the pendant hydroxyl group in its repeating unit, phenoxy exhibits proton-donor characteristics when it interacts with a second polymer that contains proton acceptors. Therefore, interactions, transreactions, or alcoholytic exchanges can increase the compatibility of phenoxy with PBA in polymer blends. Scheme 3 shows the mechanisms of the chemical exchange reactions between phenoxy and PBA. Polyesters react with a source of hydride (H^−^) ions in the phenoxy in a PBA/phenoxy blend because H^−^ is a Lewis base, or nucleophile, that attacks the δ^+^ end of the C=O bond [27,28,29]. Alcoholytic exchanges occurred between the dangling –OH of phenoxy and the carbonyl group in PBA in the heated phenoxy/PBA blends. Upon heating, chemical links are formed in the blends between the reacting phenoxy and PBA molecules. First, the exchange reaction products included hydroxyl-terminated PBA and phenoxy-grafted PBA linkages, and the extensive transreaction between PBA and phenoxy yielded a crosslinked network. The extensive transreaction in the PBA/phenoxy blend formed a crosslinked network that could not dissolve in the solvent and remained as residual solids.

### 3.5. Effect of Heat-Annealed Samples on Crystallization Behavior

**Figure 7:**
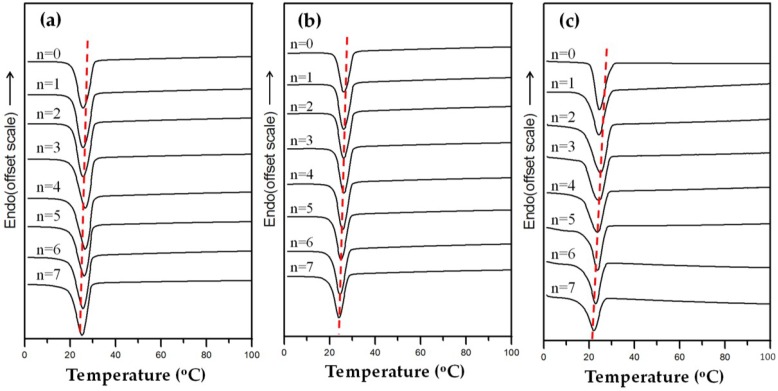
DSC thermograms of the crystallization behavior of PBA/phenoxy = 90/10 samples after annealing at 260 °C for various times then cooled in cycles of (**a**) 2 min; (**b**) 4 min; and (**c**) 6 min.

DSC was used to study the effect of the transreaction on the crystallization behavior of PBA. Figure 7 shows DSC thermograms of the crystallization of PBA/phenoxy = 90/10 samples that underwent *n* cycles of annealing between 0 and 260 °C. In Figure 7a–c, the heating rate was 10 °C/min and the annealing times (t_a_) at 260 °C were 2, 4, and 6 min, respectively. In these figures, both the exothermic heat of crystallization and the crystallization temperature (T_c_) of PBA decreased as the annealing time increased or the number of cycles of annealing at 260 °C increased. Table 2 shows the crystallization enthalpies (ΔH_c_) of PBA/phenoxy = 90/10 samples that had been annealed at 260 °C for various times then cooled (10 °C/min) cyclically. The decrease in the exothermic heat of crystallization of PBA when the total annealing time (∑t_a_) increased reveals a reduction in the number of crystalline regions of PBA. In the thermograms, both T_c_ and ΔH_c_ decreased as the transreaction proceeded, indicating that alcoholytic exchange shortened the PBA segment and reduced the number of the arrays of folded chains in the PBA segment in the copolymers. The amount of copolymer that formed in the reactive PBA/phenoxy blends affected the crystallization behavior of the crystalline polymer.

**Figure 8:**
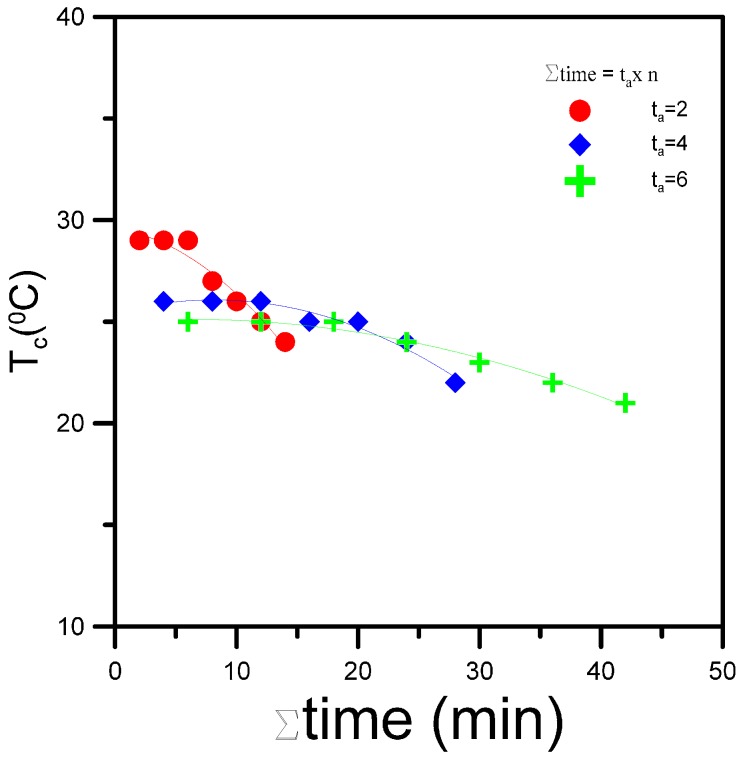
T_c_ as a function of the total annealing time for the PBA/phenoxy = 90/10 sample.

Figure 8 shows the nonisothermal melt peak temperature of crystallization (T_c_) versus the total annealing time for the PBA/phenoxy = 90/10 sample. T_c_ decreases faster as *n* increases for a given total annealing time. The results show that the degree of transreaction depends on the overall annealing time and the number, *n*, of cycles. Therefore, the copolymer content was increased stepwise. The decrease in the crystallization peak temperature reflects the extent of the exchange reactions. The behavior of T_c_ reflects a promotional exchange reaction that is caused by the new order of segments associated with the crystallization process. In this study, the complete history of annealing of the samples is considered in estimating the extent of the exchange reaction.

### 3.6. Surface Topography of Heat-Annealed Blends

**Figure 9:**
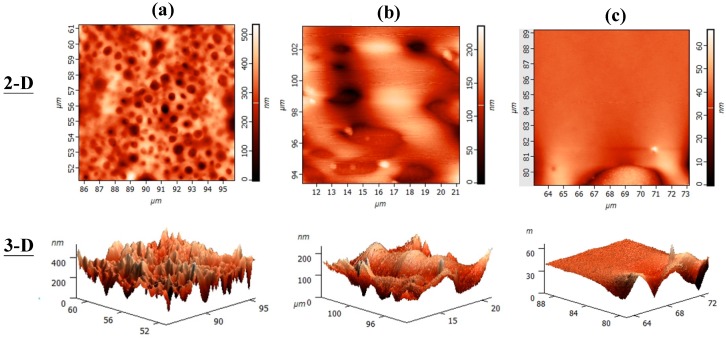
(**a**–**c**) show atomic force microscopy (AFM) height images for PBA/phenoxy (50/50) heat-annealed at 260 °C for 0, 120, and 180 min, respectively.

AFM characterization, which does not require sputter coating and preserves for the original surface morphology, was performed to obtain height profiles and other details of the surface of the samples. AFM analyses were performed to elucidate the morphology of the surface of PBA/phenoxy (50/50) samples. The various blending compositions were then exposed to an isothermal temperature of 260 °C for various times. Figure 9a–c show AFM height images of a PBA/phenoxy (50/50) sample that was heat-annealed at 260 °C for 0, 90, and 180 min. The average surface roughness values are 340, 110, and 30 nm at annealing times of 0, 120, and 180 min, respectively. The surface roughness of the blend decreased as the heat-annealing time increased, and the blend that was heated at 260 °C for 180 min had a smooth surface. The chemical exchange reactions of phenoxy with PBA proceeded between neighboring molecular chains of PBA and phenoxy. Upon heating, chemical links between the reacting phenoxy and PBA molecules were formed in the blends. This process is diffusion controlled in the melt of the blend. After annealing, the products of the exchange reaction include hydroxyl-terminated PBA, phenoxy-grafted PBA linkages, and a crosslinked network. Analysis of the surface topographies of heat-annealed blends reveals that the surface roughness depended on the products and the extent of transreaction. The surface roughness of the PBA/phenoxy blend decreased as the extent of the transreaction and the crosslinked network content increased.

## 4. Conclusions

The as-blended PBA/phenoxy sample has a homogeneous phase, a single T_g_, and a composition-dependent glass transition temperature. The interaction parameter (*χ*_12_) of the PBA/phenoxy blends, as determined by melting point depression analysis is *χ*_12_ = −0.336. Upon heat-annealing, alcoholytic exchange reactions enhanced the compatibility of blends of PBA with phenoxy. In these blends, the pendant hydroxyl group in the repeating unit of phenoxy can interact with proton-accepting carbonyl groups in the PBA. The pendant hydroxyl groups of phenoxy can participate in specific chemical interactions, or transreaction, with PBA. The carbonyl group in the original unreacted PBA chain is linked to two aliphatic linkages; however, transreactions change the linkages adjacent to the carbonyl group. This transreaction significantly changes the primary structure of polymer chains and the final properties of the blends, potentially increasing their glass transition temperatures. Upon heating, the exchange reaction products initially include hydroxyl-terminated PBA and phenoxy-grafted PBA linkages, ultimately forming a crosslinked network of PBA/phenoxy blends. Extensive transreaction in the PBA/phenoxy blend formed a crosslinked network, which did not dissolve in the solvent and formed residual solids. The leached solid sample contains polymer segments with aliphatic–(CO)–O–aromatic and aromatic–(CO)–O–aromatic structures. In this work, copolymers that were formed in the exchange reaction interrupted arrays of folded chains in the crystalline lamellae of PBA. These copolymers reduced the ordering of the polymer chain, subsequently reducing the degree of crystallization of PBA in the blends. Analysis of the surface topographies of heat-annealed blends revealed that the surface roughness depended on the products and the extent of transreaction in the PBA/phenoxy blend. Furthermore, the surface roughness of the PBA/phenoxy sample decreased as the degree of transreaction and the crosslinked network content increased.

## Figures and Tables

**Table 1:** Infrared carbonyl band with various linkages adjacent to the carbonyl group.

Ester groups	Wavenumber (cm^−1^)
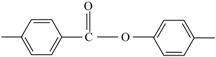	1730 cm^−1^
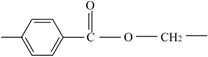	1716 cm^−1^
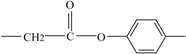	1750 cm^−1^
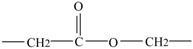	1735 cm^−1^

**Table 2:** The crystallization enthalpies for PBA/phenoxy = 90/10 samples after annealing at 260 °C for different times then cooled in cycles.

Exothermic Heat of Crystallization ΔH_n_ (J/g)	Annealing Times Per Cycle		
	2 min	4 min	6 min
ΔH*_n=_*_0_	70.9	61.2	39.6
ΔH*_n=_*_1_	65.8	57.3	34.3
ΔH*_n=_*_2_	60.8	52.2	30.2
ΔH*_n=_*_3_	55.7	49.3	15.4
ΔH*_n=_*_4_	51.6	46.2	14.8
ΔH*_n=_*_5_	49.6	43.0	14.6
ΔH*_n=_*_6_	45.4	42.4	14.4
ΔH*_n=_*_7_	44.6	40.2	12.4
